# Social Anxiety and Bullying Victimization in Children and Early Adolescents: The Role of Developmental Period and Immigrant Status

**DOI:** 10.1007/s10964-023-01865-9

**Published:** 2023-09-27

**Authors:** Nicolò Maria Iannello, Simona Caravita, Noemi Papotti, Carmen Gelati, Marina Camodeca

**Affiliations:** 1 University of Palermo, Palermo, Italy; 2 University of Stavanger, Stavanger, Norway; 3 Catholic University of Sacred Heart Brescia, Milan, Italy; 4 University of Genova, Genova, Italy; 5 University of Milano – Bicocca, Milan, Italy; 6 University of Udine, Udine, Italy

**Keywords:** Social anxiety, Childhood, Early adolescence, Immigrant students, Victimization

## Abstract

Research reveals that social anxiety may be predictive of bullying victimization, but it is not clear whether this relation stands for different groups of youth. The present study examines this association by employing a longitudinal design over 1 year and including the moderating role of developmental period (childhood vs. early adolescence) and students’ immigrant status (native vs. non-native). T1 sample included 506 children (46.44% girls, mean age *M* = 8.55 years, SD = 0.55) and 310 early adolescents (50% girls, mean age = 12.54 years, SD = 0.59) recruited in schools in Northern Italy. Due to missing cases and drop-outs from T1 to T2, the final sample comprised 443 and 203 students from primary and middle school, respectively. Social anxiety and peer victimization were assessed through self-reported questionnaires. Results indicated that victimization at T2 was predicted by a 3-way interaction between T1 social anxiety, immigrant status, and developmental period. In particular, socially anxious early adolescents with an immigrant background were the most victimized. The results are discussed in terms of group dynamics and intergroup processes. The findings highlight the importance of personal variables in the cumulation of risks: social anxiety is more predictive of bullying victimization for immigrant early adolescents than for children or native early adolescents.

## Introduction

As several studies worldwide have shown, bullying victimization is a pervasive phenomenon negatively impacting students’ life and well-being (Moore et al., 2017). Victims have been found to report mental health problems, suicidal ideations and behaviors, negative academic outcomes, increased peer rejection, and poorer school connectedness (Halliday et al., 2021; Moore et al., 2017). It is paramount to uncover possible risk factors of bullying victimization. Some studies pinpointed that an antecedent may be found in social anxiety (e.g., Ranta et al., 2013; Romera et al., 2022), defined as the tendency to experience discomfort in social interactions, to deliberately avoid social encounters that might generate distress, and to fear others’ judgments (La Greca & Stone, 1993; Tillfors et al., 2012). Although research has highlighted that socially anxious adolescents may be easy targets because of their reticent behavior (Rubin et al., 2009), the association between social anxiety and bullying victimization is not so clear for children. Possible moderating factors may also intervene and help clarify this relation, such as the developmental period and the belonging to a native or immigrant group. Using a longitudinal design, this study investigates the association between social anxiety and bullying victimization by testing to what extent it might vary as a function of students’ developmental period (childhood vs. early adolescence) and immigrant status (being native vs. having a migratory background).

### Bullying Victimization and Social Anxiety

Bullying victimization is intended as an aversive experience in which children and adolescents are regularly, intentionally, and persistently attacked by one or more peers, physically (e.g., being pushed), verbally (e.g., being teased), and relationally (e.g., being excluded) (Espelage & Swearer, 2003). Sometimes, peers may be attacked because they are perceived as deviating from what is considered normative (Thornberg, 2010), and being socially anxious may be included as a possible risk factor. Research confirms a significant positive effect of social anxiety on bullying victimization among adolescents over time (Romera et al., 2022). In a 6-months interval longitudinal study, an association between social anxiety and both traditional and cyberbullying victimization was reported among a sample of students aged 10–17 years (Pabian & Vanderbosch, 2016). A study conducted on a sample of youth from 12 to 19 years of age highlighted that social anxiety predicted increased victimization for adolescent females after 1 year (Tillfors et al., 2012). Perpetrators might attack socially anxious peers because of their shy, timid, and avoidant behavior, which, on the one hand, may make them appear weak, unable to stand up for themselves and, thus, easy target of aggression, and, on the other hand, underlines a deviation from what is considered normal according to the social expectations of being powerful, extroverted, and popular (Rubin et al., 2009; Thornberg, 2010).

Although prospective effects of social anxiety on bullying victimization, at least in short observation periods, have been so far reported among adolescents, longitudinal studies exploring this relation among children are missing. This is surprising as social anxiety might also emerge at a younger age (Gazelle & Rubin, 2010; Tillfors et al., 2012) and it can presumably expose school children to bullying victimization. In addition, it may be interesting to compare late childhood and early adolescence to elucidate whether the relation between social anxiety and bullying victimization might vary at specific points in development, usually coinciding with school transitions.

### The Role of Developmental Period

Researchers commonly asserted that social anxiety reaches its peak during early adolescence and that this trend might be attributed to the critical bodily, cognitive, and social challenges marking this phase of life (Tillfors et al., 2012; Tillfors & Van Zalk, 2015). With regards to the physical transformations, morphological changes provoked by pubertal maturation might enhance social anxiety among adolescents who might feel uncomfortable with their bodies and worried about others’ judgements (La Greca & Ranta, 2015). Advancements in social cognitions as well might contribute to youth’s enhanced social anxiety, making early adolescents more prone to compare themselves to others and more concerned about peer evaluations in several situations than younger children (Parker et al., 2006).

As to social changes, early adolescence usually coincides with the transition to middle/ secondary school, more requesting than primary school, and where youth are confronted with larger social environments, new approaches to teaching and learning, teachers’ higher expectations, and challenges in forming and managing new friendships and relationships (Jindal-Snape & Miller, 2008). All these challenges can presumably impact students’ psychological well-being and favor fear and distress associated with social situations and interactions (Grills-Taquechel et al., 2010). In addition to that, it is worth bearing in mind that withdrawn attitudes might be more salient and negatively evaluated by peers as they grow up (Ladd, 2006; Rubin et al., 2009). In early adolescence, when expanding friendships and participating in peer activities are thought to be normative, inhibited, and avoidant students are not considered suitable for social cliques by some schoolmates, who might see them as eligible for victimization (Rubin et al., 2009). Indeed, also bullying victimization peaks during middle school years (Nylund et al., 2007), probably because early adolescents might be more prone to positively value attacking their peers to gain power within their expanding social networks (Pellegrini & Long, 2002). In line with these developmental considerations, it is likely that the association between social anxiety and bullying victimization is stronger for early adolescents than for late children.

### The Role of Immigrant Status

A growing body of research pointed out that being an immigrant may be considered a risk factor for bullying victimization (Maynard et al., 2016; Xu et al., 2020). Although ethnicity alone, as a demographic variable, was not strongly related to victimization (Vitoroulis & Vaillancourt, 2015), several factors associated with immigration may account for it, such as classroom and school ethnic diversity (Basilici et al., 2022), acculturation stress (Messinger et al., 2012), adverse contextual aspects (Xu et al., 2020), or prejudice (Caravita et al., 2020; Iannello et al., 2021). Social anxiety might be of particular concern for non-native children and adolescents because it is associated with the stress related to the inclusion in a new culture. Students with an immigrant background navigate between two cultures and need to cope with the belongingness to a minority group; they may experience discomfort in social relationships because of the differences between their and majority group’s language, norms, beliefs, and traditions (Berry et al., 2006), which can be the basis for prejudice and discriminations. Experiences of discrimination may amplify immigrants’ social anxiety (Doğan & Strohmeier, 2020) and lead to anxious anticipations of possible negative consequences related to connecting with peers; immigrant children and youth are likely to develop a negative appraisal of social situations and to disengage from interactions with others to avoid real or imagined refusal or disapproval (Xu et al., 2021). In addition, immigrants’ social anxiety may emerge from their perceived lower social status and social self-efficacy, and from difficulties to conform to social standards of host society (Hsu et al., 2012).

From a developmental perspective, having an immigrant background poses additional challenges to early adolescents and might further interfere with the developmental tasks which are typical of this period (Jugert & Titzmann, 2020). Furthermore, as already advanced, with age students become more prone to reject those schoolmates who do not conform to the social expectations of being extroverted and popular (Ladd, 2006; Rubin et al., 2009), particularly in a less controlled environment such as middle school classrooms. In their daily interaction with others, socially anxious early adolescents with an immigrant background might be perceived as not fitting in with the peer group for both their ethnicity and their social anxiety to a greater extent than their younger counterparts. In other words, inhibited immigrant adolescents are likely to be perceived as doubly “deviant” by classmates, who might turn to aggression against them to preserve their group identity and functioning (Park & Killen, 2010; Rutland et al., 2010). On the base of these considerations, immigrant status might be an additional risk strengthening the association between social anxiety and bullying victimization, in particular for early adolescents.

### Possible Covariates: Gender and Covid-19 Impact

Although beyond the scope of this work and without advancing any hypotheses, gender and the impact of Covid-19 were considered important covariates to control. Gender may affect social anxiety, bullying victimization, and their relations. Even if women are more likely to exhibit social anxiety disorder than men, especially during adolescence (Asher et al., 2017), both boys and girls have similar levels of social anxiety, and boys report more general social avoidance and distress than girls (Ranta et al., 2012). With regards to bullying victimization, it seems that girls are generally less victimized than boys (de Bruyn et al., 2010), but this prevalence is not universal (Smith et al., 2019). Finally, some studies showed that social anxiety was related to victimization for boys (Erath et al., 2007), whereas others found that it predicted increases in victimization for females only (Tillfors et al., 2012).

Since data of this study were collected before and after the Covid-19 pandemic outbreak, it was proper to consider whether this experience and the related lockdowns and social distancing might have affected the level of distress in some students and influenced the quality of their peer relationships (Cowie & Myers, 2021). A recent review of the literature indicated that, during the pandemic, social anxiety has increased among young populations, but the lockdowns might have also offered relief to adolescents with high social anxiety prior to the pandemic (Kindred & Bates, 2023). Studies on the impact of the Covid-19 on bullying victimization seem to report controversial findings, with some of them highlighting a decrease in bullying episodes (Vaillancourt et al., 2021), and others showing an increased prevalence (Forsberg & Thorvaldsen, 2022). Considering these findings, it seemed useful to control the impact of gender and the pandemic in the analyses.

## The Present Study

Social anxiety has been found to be a risk factor contributing to bullying victimization in adolescence, but research investigating this association in children is missing. It is also unclear whether the strength of this relation is different for native and immigrant students. This study aimed to investigate the longitudinal association between social anxiety and bullying victimization in different groups of youth. Based on the above mentioned theoretical and empirical background, it was hypothesized that social anxiety predicted bullying victimization over 1-year time. It also was expected that this association was stronger among early adolescents (attending middle school) than among late children (attending primary school) and among immigrant than native (Italian) students. Lastly, the association between social anxiety and bullying victimization was expected to be stronger for immigrant adolescents than for children or Italian adolescents.

## Method

### Participants

The final sample present at T1 included 506 children (271 boys and 235 girls, aged 7–10 years, *M* = 8.55 years, SD = 0.55) attending the third and fourth grade of 10 primary schools, and 310 early adolescents (155 boys and 155 girls, aged 11–15 years, *M* = 12.54 years, SD = 0.59) attending the first two grades of a middle school in Northern Italy.

Among students present at T1, 472 (93.28%) children and 234 (75.8%) early adolescents were also present at T2. No differences emerged in bullying victimization and social anxiety between these participants and those who dropped, although a higher number of immigrant students dropped in comparison with Italian students (*χ*^2^ = 4.64*; p* = 0.03). In general, students did not participate after 1 year because they moved to another school or were absent in the day of data collection. Thus, at T2, the total sample consisted of 706 students. It was used the mean imputation method with SPSS when missing values were less than 10% in each scale, after verifying that the missing data were random (Rubin, 1976). However, 58 participants (7.22%; 27 children and 31 early adolescents) had more than 10% of missing data in any of the study variables, so it was not possible to carry out mean imputation and they were removed from the database. Two participants did not indicate their origin and could not be assigned the Italian or immigrant status. The final analyses were conducted on 443 children and 203 early adolescents.

Of these participants, 476 (73.7%) were Italian students (at least one parent was born in Italy) and 170 (26.3%; 28% in primary school and 26.3% in middle school) were first- and second-generation immigrants (both parents were born abroad), which mirrors the proportion Italian/ immigrants in the considered geographical areas. Specifically, the cultural composition of this latter subsample was as follows: 26.1% from Eastern Europe, 22.6% from North Africa, 18.3% from Asia, 11.3% from Central and South America, and the remaining 21.7% from other states of Africa, Europe, Oceania, and the Middle East.

### Procedure

This study is a part of a larger project presented to school principals and teachers, who approved and accepted to participate. Parents were sent a letter to explain the research and were asked active consent for the participation of their children, which was given by 84.4 and 79.5% of the contacted families of primary and middle school students, respectively. The research was conducted according to the Helsinki Declaration and was approved by the ethic committees of the University of Udine and the Catholic University of Brescia (Italy).

Bullying victimization and social anxiety were assessed at both time points, whereas the Covid-19 impact was assessed at T2. Data were always collected in classroom. At T1 (January–February 2020), a trained researcher was present, who provided information, answered questions, and administered the questionnaires in paper format (primary school) and online using the Qualtrics platform (middle school). Between the first and the second data collections, the Covid-19 pandemic happened. During the early stages of the pandemic, Italy faced a 3-months lockdown and from March 2020 to June 2020 (the end of the school year) all students were forced to distance learning and did not have the opportunity to meet with each other. Following the timeline of a larger project and considering the availability of schools, data were collected again after 12–15 months (February–May 2021, T2), when all students had the opportunity to go to school again in presence, meet their classmates, and re-establish their relational networks. Due to pandemic restrictions and difficulties in entering schools for external personnel at T2, researchers trained primary school teachers online to administer paper questionnaires and assured their availability to be contacted by phone or video call during administration in case of need. In middle school, students still filled the questionnaires on the Qualtrics platform in their classrooms, but one researcher was always present online.

### Measures

#### Bullying victimization

The subscale of victimization of the Florence Bullying and Victimization Scales (Palladino et al., 2016) was used, after providing students with a definition of bullying: *“We say it is bullying when some students say mean things to their peers, make fun of or offend them, when they deliberately ignore or exclude them from a group, when they hit, kick, or threat them, or badmouth behind their back. These and similar things may happen often, and victims cannot defend themselves. It is always bullying when a student is teased frequently and meanly; it is not bullying when two peers of the same strength quarrel or fight with each other”*. Afterwards, students were asked to respond to 7 items about the frequency of the experienced physical, verbal, and indirect victimization, during the last 2–3 months (e.g., “In the last 2–3 months I have been mocked”). Each item was rated on a 5-point Likert Scale (from 1 = *never* to 5 = *several times a week*). The scale was calculated as the average score across the seven items; it showed good reliability, evaluated by Cronbach’s alpha (T1 = 0.83; T2 = 0.82).

#### Social anxiety

The Social Anxiety Scale (La Greca & Stone, 1993; Italian adaptation for children and adolescents by Caravita & Camodeca, 2019) was used to assess fear, worry, withdrawal, and avoidance in the context of peer relations. It consists of 14 items (e.g., “I worry about what other kids think of me”; “I get nervous talking to new kids”), with a 5-points Likert answer modality (from 1 = *not at all* to 5 = *all the time*). Cronbach’s alphas were 0.86 and 0.91 (at T1 and T2, respectively).

#### Covid-19 impact

An adaptation of the Children’s Revised Impact of Event Scale (CRIES-8; Perrin et al., 2005; Italian version by Davico et al., 2021) was employed to assess the impact that Covid-19 had on students in terms of intrusive thinking and avoidance. It comprises 8 items (e.g., “I think about it when I don’t want”; “I try to forget it”). The preliminary introduction was rephrased to make it more suitable for young children: “*Now you will read a list of things that could happen to children/teens when they meet a difficult event like the one we are experiencing now because of the Coronavirus. Think about how you feel about the Covid-19, the lockdown, and everything that’s going on right now”*. Participants had to answer on a 4-point Likert scale (from 0 = *never* to 3 = *often*). The Cronbach’s alpha was 0.75 (T2 only).

### Statistical Analyses

Preliminary analyses, conducted with SPSS 27, included descriptive statistics, *t* tests to investigate differences in gender, immigrant status, and developmental period, and correlations among study variables.

To test whether social anxiety at T1 was associated with bullying victimization at T2 and whether this association was moderated by developmental period and immigrant status, a cross-lagged model of the autoregressive and cross lagged paths of social anxiety and victimization from T1 to T2 was run (MPlus 8.4; Muthén & Muthén, 1998–2017). In the model, gender, developmental period, immigrant status, and the impact of Covid-19 were included among predictors of both victimization and social anxiety at T2, and 2-way and 3-way interactions of social anxiety (centered scores), developmental period, and immigrant status among predictors of victimization at T2 (2-way interaction terms: social anxiety*developmental period, social anxiety*immigrant status, immigrant status*developmental period; 3-way interaction term: social anxiety*developmental period *immigrant status). Before running the cross-lagged model, the measurement invariance across time of the social anxiety and the victimization measures was tested (loadings and intercepts of the indicators constrained to be equal across the two-time points; MLR robust estimator). Then paths models were run to examine the autoregressive associations of social anxiety and victimization between T1 and T2. Lastly, the cross-lagged model was run, including covariates and interaction terms, as a path analysis model. To examine significant interactions, separate and multigroup models for the developmental period and immigrant status groups were run as follow-up analyses. To test the autoregressive and cross-lagged models, Bayesian estimation (1000 iterations) was used, in which the default is using two independent Markov chain Monte Carlo chains (Muthén & Muthén, 1998–2017). In all the tested models, the cluster effects of belonging to classrooms were controlled by using the Complex option available in MPlus. It was tested the model goodness of fit examining the model Chi-square index, which needs to be non-significant for well-fitting models. As the Chi-square index is sensitive to the sample size, it was proper to also consider the comparative fit index (CFI) and the Tucker–Lewis index (TLI), which need to be equal or superior to 0.90 for an acceptable model fit (Hu & Bentler, 1998). Lastly, the root mean square error of approximation (RMSEA) and the standardized root mean square residual index (SRMR) were examined to estimate the residuals of the non-explained data. These residual indices need to be equal or lower than 0.08 for an acceptable model fit (Hu & Bentler, 1998).

## Results

### Preliminary Analyses

The descriptive statistics of the research variables and *t* tests are displayed in Table 1. *T* tests indicate that, both at T1 and T2, high levels of bullying victimization were associated with immigrant status and with being younger. Early adolescents were more socially anxious at T1 and less affected by Covid-19 than children. Girls were more socially anxious than boys at both points of time.

**Table 1:** Means and standard deviations of study variables, and *T* tests

	*M* (SD) Total sample	*M* (SD) Gender		*t* test Gender^a^	*M* (SD) Immigrant status		*t* test Immigrant status^b^	*M* (SD) Developmental period		*t* test Developmental period^c^
		Boys	Girls		Immigrant	Italian		Children	Early adolescents	
Victimization T1	1.43 (0.55)	1.46 (0.54)	1.40 (0.55)	1.55*; p* = 0.12	1.51 (0.67)	1.40 (0.50)	−2.20*; p* = 0.03	1.50 (0.57)	1.27 (0.46)	5.05*; p* < 0.001
Victimization T2	1.36 (0.48)	1.39 (0.50)	1.33 (0.45)	1.61*; p* = 0.11	1.47 (0.60)	1.32 (0.42)	−3.38*; p* < 0.001	1.42 (0.38)	1.23 (0.72)	4.90*; p* < 0.001
Social Anxiety T1	2.18 (0.76)	2.05 (0.72)	2.31 (0.78)	−4.43*; p* < 0.001	2.26 (0.87)	2.15 (0.72)	−1.69*; p* = 0.09	2.11 (0.72)	2.31 (0.83)	−3.21*; p* = 0.001
Social Anxiety T2	2.26 (0.89)	2.06 (0.79)	2.48 (0.95)	−6.11; *p* < 0.001	2.24 (0.90)	2.27 (0.89)	0.41; *p* = 0.68	2.23 (0.86)	2.33 (0.95)	−1.25; *p* = 0.21
Covid-19 Impact T2	1.43 (0.69)	1.39 (0.72)	1.46 (0.64)	−1.42*; p* = 0.16	1.39 (0.71)	1.43 (0.68)	0.66*; p* = 0.51	1.53 (0.69)	1.20 (0.63)	5.84*; p* < 0.001

^a^gdf = 3.651

^b^gdf = 1.651

^c^gdf = 1.653

Correlations (Table 2) indicate a positive association between social anxiety and victimization both at T1 and at T2 and between the impact of the Covid-19 and all the other variables. Victimization at T1 and T2 and social anxiety at T1 and T2 correlated with each other.

**Table 2:** Correlations among study variables

	Victimization (T1)	Victimization (T2)	Social Anxiety (T1)	Social Anxiety (T2)
Victimization (T2)	0.45**			
Social Anxiety (T1)	0.34**	0.19**		
Social Anxiety (T2)	0.23**	0.30**	0.45**	
Covid-19 Impact (T2)	0.16**	0.22**	0.08*	0.24**

* *p* < 0.05. ** p < 0.01

### Cross-Lagged Model

As first step, the measurement invariance across time of the scales assessing bullying victimization and social anxiety was tested. For the social anxiety scale (La Greca & Stone, 1993), parcel scores of the 14 items included in its subscales were used (Caravita & Camodeca, 2019). The scalar invariance, with loadings and intercepts of the indicators as equal across time, was confirmed for both the victimization scale (after correlating the errors of two items: Chi-square(87) = 222.006, *p* = 0.000, CFI = 0.923, TLI = 0.932, RMSEA = 0.049, CI = 0.041–0.057, SRMS = 0.058) and the social anxiety scale (after correlating the errors of two parcels out of six: Chi-square(56) = 268.937, *p* = 0.000, CFI = 0.942, *TLI* = 0.932, RMSEA = 0.077, *CI* = 0.068–0.086, SRMS = 0.055).

When the autoregressive paths of social anxiety and victimization were tested, the model fitted the data well (Chi-square(2) = 7.623, *p* = 0.0221, CFI = 0.976, TLI = 0.941, RMSEA = 0.066, *CI* = 0.021–0.118, SRMR = 0.036). The two autoregressive paths were positive and significant for both victimization (beta = 0.445, *p* = 0.000) and social anxiety (beta = 0.443, *p* = 0.000).

**Fig. 1:**
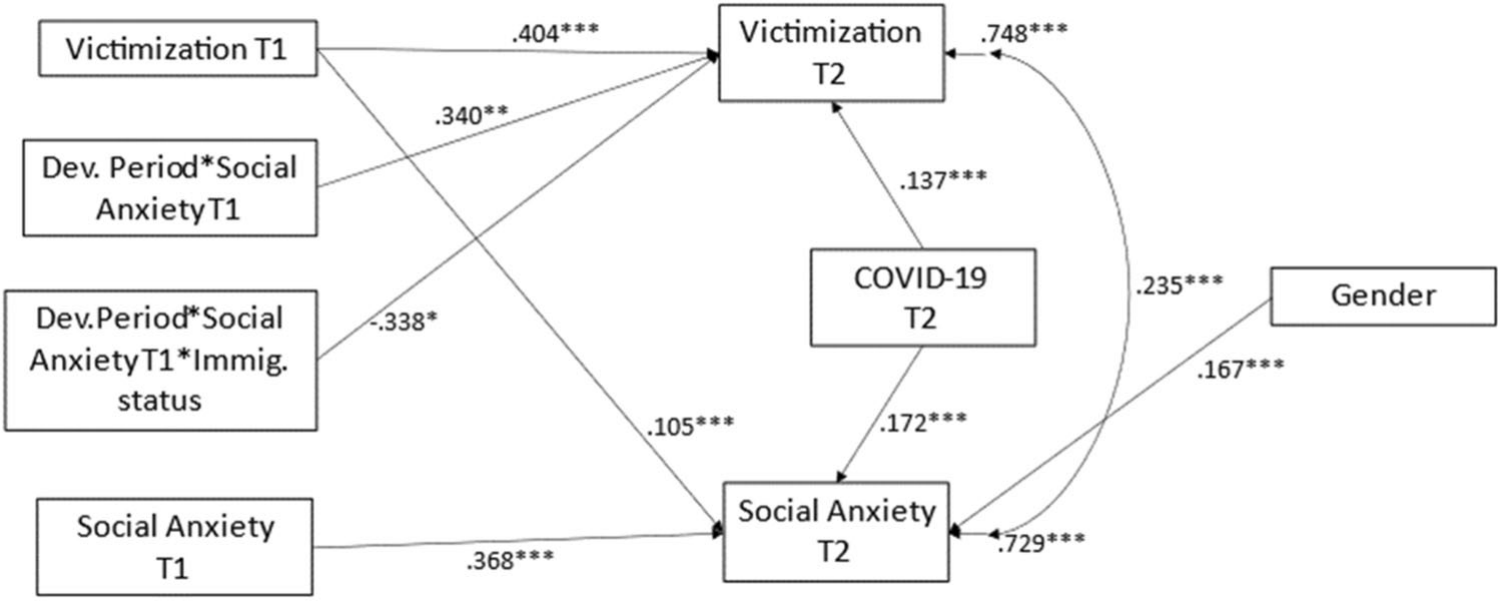
Cross-lagged model of social anxiety and bullying victimization, with covariates and moderation terms. Note. Only significant paths are displayed. *p < 0.05, **p < 0.01, ***p < 0.001

The cross-lagged model of the associations between victimization and social anxiety, including gender, COVID-19 impact, immigrant status, and developmental period as covariates for both victimization and social anxiety at T2, and 2-way and 3-way interaction terms of social anxiety at T1 by developmental period and by immigrant status as predictors of victimization at T2 fitted the data well: Chi-square(4) = 2.909, *p* = 0.5732, CFI = 1.000, TLI = 1.000, RMSEA = 0.000, *CI* = 0.000–0.051, SRMR = 0.012. The significant associations among the variables are displayed in Fig. 1.

The paths explained 25% of the variance (*R*^*2*^ = 0.252, *p* = 0.000) of victimization at T2, and the 27% of the variance of social anxiety at T2 (*R*^*2*^ = 0.271, *p* = 0.000). Social anxiety at T2 was predicted by social anxiety at T1 (beta = 0.368, *p* = 0.000), victimization at T1 (beta = 0.105, *p* = 0.000), being female (beta = 0.167, *p* = 0.000), and impact of COVID-19 (beta = 0.172, *p* = 0.000). Victimization at T2 was associated with victimization at T1 (beta = 0.404, *p* = 0.000), Covid-19 impact (beta = 0.137, *p* = 0.001), the interaction between T1 social anxiety and developmental period (beta = 0.0340, *p* = 0.010), as well as the 3-way interaction between T1 social anxiety, developmental period, and immigrant status (beta = −0.338, *p* = 0.012).

To interpret the interaction effect, first separate models for primary school children (Chi-square(1) = 1.302, *p* = 0.2539, CFI = 0.999, TLI = 0.981, RMSEA = 0.026, CI = 0.000–0.132, SRMR = 0.003) and middle school adolescents (Chi-square(1) = 0.158, *p* = 0.6907, CFI = 1.000, TLI = 1.000, RMSEA = 0.000, *CI* = 0.000–0.138, SRMR = 0.002) were run. Associations among variables are reported in Table 3. Among children, social anxiety at T2 (*R*^*2*^ = 0.256, *p* = 0.000) was predicted positively by social anxiety at T1, being female, and COVID-19 impact, while victimization at T2 (*R*^*2*^ = 0.227, *p* = 0.000) was significantly predicted by victimization at T1 and COVID-19 impact, but neither by social anxiety nor by the interaction term social anxiety by immigrant status. Among secondary school adolescents, social anxiety at T2 (*R*^*2*^ = 0.299, *p* = 0.000) was positively predicted by the autoregressive path, being victimized at T1, being female, and the COVID-19 impact, while victimization at T2 (*R*^*2*^ = 0.243, *p* = 0.001) was predicted by the autoregressive path, social anxiety at T1 and the 2-way interaction between social anxiety at T1 and immigrant status (beta = −0.394, *p* = 0.004).

**Table 3:** Associations of the cross-lagged model among primary school children and middle school early adolescents, with T2 victimization and T2 social anxiety as outcome variables

	Children		Early Adolescents	
	Beta	*p*	Beta	*p*
*Victimization T2*				
Gender (0 = Boy; 1 = Girl)	−0.052	>0.05	−0.024	>0.05
Immigrant status (0 = Italian; 1 = Immigrant)	−0.060	>0.05	−0.090	>0.05
Victimization T1	0.398	0.000	0.426	0.000
Social Anxiety T1	−0.203	>0.05	0.493	0.001
Covid-19 Impact T2	0.174	0.000	0.008	>0.05
Immigrant Status x Social Anxiety	0.229	>0.05	−0.394	0.004
*Social Anxiety T2*				
Gender (0 = Boy; 1 = Girl)	0.166	0.000	0.172	0.000
Immigrant status (0 = Italian; 1 = Immigrant)	0.033	>0.05	0.097	>0.05
Victimization T1	0.115	>0.05	0.084	0.040
Social Anxiety T1	0.331	0.000	0.410	0.000
Covid-19 Impact T2	0.193	0.000	0.121	0.000

To interpret this last interaction, corresponding to the 3-way interaction of the cross-lagged model, a multigroup model was run among the secondary school adolescents, comparing the students with Italian heritage and the students with migratory background, specifying the autoregressive and the cross-lagged paths for social anxiety and victimization, which were both predicted by gender and COVID-19 impact. The model was saturated (no degrees of freedom, with no estimation of the goodness of fit); the associations among the variables are reported in Table 4. Social anxiety at T1 predicted victimization at T2 only among the students with migratory background (beta = 0.320, *p* = 0.036) and it was the only significant predictor of victimization at T2 for this student group (*R*^*2*^ = 0.256, *p* = 0.015), while the autoregressive path was the only significant predictor of social anxiety at T2 (*R*^*2*^ = 0.370, *p* = 0.001). Among the adolescents of Italian heritage, victimization at T2 (*R*^*2*^ = 0.238, *p* = 0.003) was significantly predicted only by the autoregressive path, while social anxiety at T2 (*R*^*2*^ = 0.264, *p* = 0.000) was predicted by the autoregressive path, gender (being female), and the COVID-19 impact.

**Table 4:** Associations of the cross-lagged model among middle school early adolescents with Italian heritage and migratory background, with T2 victimization and T2 social anxiety as outcome variables

	Italians		Immigrants	
	Beta	*p*	Beta	*p*
*Victimization T2*				
Gender (0 = Boy; 1 = Girl)	0.006	>0.05	−0.129	>0.05
Victimization T1	0.479	0.000	0.260	>0.05
Social Anxiety T1	0.030	>0.05	0.320	0.036
Covid-19 Impact T2	−0.015	>0.05	0.105	>0.05
*Social Anxiety T2*				
Gender (0 = Boy; 1 = Girl)	0.161	0.004	0.222	>0.05
Victimization T1	0.072	>0.05	0.143	>0.05
Social Anxiety T1	0.406	0.000	0.408	0.009
Covid-19 Impact T2	0.117	0.002	0.096	>0.05

These results were also confirmed in sensitivity analyses testing moderated moderation models using PROCESS module in SPSS. Furthermore, even if going beyond the scope of this study, alternative cross-lagged models were run to test the possibility that the developmental period and the immigrant status also moderated the path from victimization at T1 to social anxiety at T2. In none of these models, this association was moderated, while the 3-way interaction between social anxiety, developmental period, and immigrant status continued to significantly predict victimization at T2.

## Discussion

Although research suggested a possible longitudinal association between social anxiety and bullying victimization among adolescent populations (Pabian & Vandebosch, 2016; Romera et al., 2022), a focus on children was missing, as well as an investigation on possible individual moderators, such as students’ developmental period and immigrant status (Kingery et al., 2010). The current study proposed a model examining whether the longitudinal association between social anxiety and bullying victimization varied between children and early adolescents and between immigrants and native students. Findings confirmed the association for early adolescents, but not for children, and indicated that it was mostly significant when they had an immigrant background. In the following paragraphs, the outcomes of this study are discussed thoroughly.

The hypothesis that social anxiety predicted bullying victimization over time was not supported without moderation, underlining the need of considering intervening variables. Although it was not among the hypotheses, results also revealed an inverse relationship from bullying victimization to social anxiety after 1 year, supporting previous studies (e.g., Ranta et al., 2013; Siegel et al., 2009). It is feasible to assume that children and adolescents who are repeatedly harassed may feel constantly under pressure, think that they are back mouthed, and wish to avoid social situations, eventually developing a sort of fear in interacting with peers.

When comparing two developmental periods, the longitudinal association between social anxiety and bullying victimization was found for adolescents and not for children, as expected. It could be argued that apprehension and worry about others’ judgements and discomfort in social encounters might lead children to avoid contacts with other schoolmates; these limited occasions for interactions might prevent them from being visible and, thus, from being attacked (Storch et al., 2005). It is possible that occasions of bullying victimization decreased during and just after the pandemic, and that children who were anxious before the pandemic could have been less victimized when back at school again. In contrast, early adolescents may feel more pressure to interact with others and may have more difficulties in avoiding social contacts, which may put socially anxious youth at risk of victimization (Parker et al., 2006). Even during the pandemic, it is feasible that adolescents, compared with children, were more easily involved in contacts with schoolmates or more easily reachable by them, either for their higher autonomy or for a greater access to social digital means.

Other reasons may be advanced to explain why socially anxious adolescents were more victimized than children. Early adolescence is a period of physical, cognitive, social, and affective changes, which is accompanied by an increased awareness of oneself and others. It is likely that sometimes youth feel discomfort, think that they are wrong or that everyone is judging them, which make them withdrawn, reticent, and scared in social situations. They may be harassed or excluded by peers, who, at this age, place a great value on the belongingness to popular cliques and set strict boundaries against those who deviate from the standards (Gavin & Furman, 1989) of being self-confident, assertive, and fearless. An avoidant behavior is a salient characteristic that youth may select to reject those who do not fit in with the peer group (Rubin et al., 2009).

The impression of not fitting with the group standards is even more evident if socially anxious adolescents have an immigrant background. In line with an acculturative stress framework (Berry et al., 2006), it is argued that the difficulties that immigrant students may experience in adapting to a different culture could affect their psychological well-being and social adjustment. For instance, immigrant youth may be marked with social stigma (e.g., being less fluent in language, having different aspect, customs, and traditions), which may weaken their self-efficacy, increase their distress in social interactions (e.g., they may feel judged, unaccepted, and uncomfortable), and, eventually, push them to keep away from peers. This type of behavior, however, might reinforce schoolmates’ beliefs that these students are unpopular, odd, weak and, thus, easy targets of aggression.

Early adolescents with an immigrant background are also likely to have a longer history of discrimination and to be more aware of its different forms compared to immigrant children (Brown & Bigler, 2005). They may have developed higher sensitiveness and recognition of threatening cues, which contribute to their fear of being mistreated and to a possible misinterpretation of their social interactions as prejudiced or hostile (Xu et al., 2021). These interpretations may condition immigrant youth’s behaviors and lead them to be avoidant and inhibited, which, in the eyes of peers, results in appearing less desirable, more isolated and, thus, more vulnerable to victimization (Siegel et al., 2009).

Considering group dynamics and intergroup processes among adolescents might also shed some light on the current findings (Park & Killen, 2010; Rutland et al., 2010). Early adolescence is a sensitive time to explore one’s own social and ethnic identity, to form prejudices, and to differentiate between members of the in-group and members of the out-group (Degner & Wentura, 2010). Youth are likely to interpret their social world through the lens of the “Us” vs. “Them” construal, which fosters polarized social categories (e.g., normal/odd, powerful/weak, Italian/non-Italian), in-group favoritism, and out-group derogation (Tajfel et al., 1971). As social categories and prejudices have been found to negatively affect the quality of intergroup relations (Kawakami et al., 2017), it is likely that they may motivate and justify aggression against those who are not accepted as members of one’s cliques because of some specific behaviors, personal traits, or ethnic background (Caravita et al., 2020). Overall considered, it seemed that social anxiety, early adolescence, and immigrant status might be regarded as cumulative risk factors enhancing the likelihood of being victimized in bullying situations.

As a final note on covariates, gender was not associated with bullying victimization. Although this is not a novel finding in research, it might be due to the fact that direct and indirect forms of victimization were not distinguished; future research should differentiate between them. In line with the literature, girls reported a higher level of social anxiety than boys (Asher et al., 2017). Regarding the impact of Covid-19, findings highlighted a stronger effect of the pandemic on children than on early adolescents, maybe because teens are more able to rationalize and overcome intrusive thoughts than children, or because they more easily find distracting activities. In any case, irrespective of the developmental period, the impact of Covid-19 was correlated with T1 and T2 bullying victimization and social anxiety, which, according to a cumulative risk hypothesis, may indicate a higher vulnerability for those who already showed precedent fragilities. In a vicious circle, victimized and socially anxious students may develop a higher stress due to the pandemic, but, also, being more concerned with the pandemic may be a risk condition heightening worry for social contact and tendency to withdraw, and weakening social bonds and coping strategies. Being beyond the scope of the present work, this issue was not investigated further.

This study should be interpreted in light of several shortcomings. All the measures were self-reported, which may have led to a social desirability bias and to outcomes influenced by personal interpretations. Future research should adopt a multi-informant approach (e.g., using peer reports) to obtain a clearer picture of children and youth’s social anxiety and quality of peer relationships. The instrument employed for detecting bullying victimization did not allow a distinction between direct and indirect forms, which might have helped capturing a more nuanced picture of the association between social anxiety and bullying victimization (Ranta et al., 2013; Siegel et al., 2009). Given that this study was conducted before and after the Covid-19 outbreak, it is possible that, due to the considerable amount of time spent online during the pandemic, social anxiety might have increased forms of harassment different from the face-to-face victimization, such as the cyber-victimization, that was not considered in this contribution. Another limitation is that this study did not distinguish between first and second generation immigrants, mainly because first generation youth were very few; it would be useful to investigate whether the association between social anxiety and bullying victimization works similarly for children and youth with a recent history of immigration and for those who were born in the country of settlement (Doğan & Strohmeier, 2020; Strohmeier et al., 2011).

The current study has some strengths that should be mentioned as well. The longitudinal design and the large size of the sample including students with different ages and cultural backgrounds are among them. The study is one of the first investigating the topic of social anxiety in the context of immigration, by combining an acculturative stress framework and a developmental standpoint.

Results suggest implications for teachers, educators, and practitioners regarding the implementation of intervention programs. Interventions should be aimed at identifying immigrant students’ needs concerning both their development and acculturation processes (Motti-Stefanidi et al., 2015), as socially anxious immigrants may be mostly vulnerable. This type of interventions may be particularly useful for those immigrants in the pivotal phase of adolescence. In addition, interventions are recommended to promote an inclusive school climate enhancing youths’ psychosocial adjustment, the quality of social relationships, care for fragile peers, understanding and acceptance of withdrawn behavior, and intergroup contact, which may foster mutual knowledge and trust (Inguglia et al., 2020).

## Conclusion

Although social anxiety has been considered a possible antecedent of bullying victimization, longitudinal data are still scarce, and possible moderators, such as developmental period and immigrant status, had not been considered yet. Employing a longitudinal design and a sample of native and immigrant children and adolescents, this study highlights that being an early adolescent with a migratory background is a risky condition that cumulates with social anxiety in predicting bullying victimization. The outcomes contribute to enhance knowledge on early adolescence, providing evidence that, in this complex period, social and psychological issues are amplified and not always perfectly managed, in particular if youth have an immigrant background.
